# Impact of Extrusion Parameters on the Formation of *N^ε^*-(Carboxymethyl)lysine, *N^ε^*-(Carboxyethyl)lysine and Acrylamide in Plant-Based Meat Analogues

**DOI:** 10.3390/ijms25168668

**Published:** 2024-08-08

**Authors:** Yurong Ma, Shuang Fu, Ka-Wing Cheng, Bin Liu

**Affiliations:** 1 College of Chemistry and Environmental Engineering, Shenzhen University, Shenzhen 518060, China; yurongm@163.com (Y.M.); fushuang0322@163.com (S.F.); kwcheng@szu.edu.cn (K.-W.C.); 2 Shenzhen Key Laboratory of Food Nutrition and Health, Shenzhen University, Shenzhen 518060, China; 3 Institute for Innovative Development of Food Industry, Shenzhen University, Shenzhen 518060, China

**Keywords:** advanced glycation end-products, acrylamide, plant-based meat analogue, extrusion, parameter

## Abstract

To investigate the impact of extrusion parameters on the formation of *N^ε^*-(carboxymethyl)lysine (CML), *N^ε^*-(carboxyethyl)lysine (CEL) and acrylamide in plant-based meat analogues (PBMAs), the content changes and the correlations of compounds related to their formation were studied. The extrusion promoted CML, CEL and acrylamide formation, with more CEL being formed than CML. Variations in the moisture level and barrel temperature exerted a greater influence on the CML, CEL, acrylamide and α-dicarbonyl compounds than the screw speed and the feed rate. An increase in the moisture content led to a decrease in the CEL content, whereas it enhanced CML formation. The impact of moisture on acrylamide formation varied depending on whether low- or high-moisture extrusion was applied. Elevated temperatures promoted the accumulation of CEL, methylglyoxal and 2,3-butanedione while diminishing the accumulation of CML, acrylamide, glyoxal and 3-deoxyglucosone. CML and CEL were positively correlated with glyoxal and methylglyoxal, respectively. CEL and methylglyoxal were negatively correlated with protein and water content, whereas CML, glyoxal and 3-deoxyglucosone displayed positive correlations. In summary, higher moisture levels and feed rates and lower screw speeds and barrel temperatures are advantageous for producing PBMAs with lower CEL and total advanced glycation end-products contents, while lower or higher moisture contents, a lower feed rate and a higher barrel temperature are beneficial to reducing the acrylamide content.

## 1. Introduction

Advanced glycation end-products (AGEs) are a group of compounds formed through Maillard reactions and lipid peroxidation [1]. *N^ε^*-(carboxymethyl)lysine (CML) and *N^ε^*-(carboxyethyl)lysine (CEL) are the most extensively studied AGEs, and they are considered biomarkers of AGEs. They are generated not only from the degradation of fructoselysine (an Amadori product) but also from the reaction of α-dicarbonyl compounds (such as methylglyoxal and glyoxal) with lysine and arginine residues [2,3]. AGEs are related to various diseases, including diabetes, atherosclerosis and neurodegenerative diseases [4]. Dietary AGEs and endogenous AGEs are the main sources of AGEs in the human body, with foods being the predominant contributors [5,6]. Approximately 10% of dietary AGEs can be absorbed by humans, of which 70 and 95% cannot be excreted but rather remain in the bodies of individuals with normal renal function and severe renal disease, respectively [7]. After oral ingestion, protein-bound AGEs are subject to digestion by gastrointestinal enzymes. Free AGEs permeate intestinal cells through simple diffusion, whereas peptide-bound AGEs are transported into intestinal cells via peptide transporter PEPT1 or through mechanisms such as paracellular transport, transcytosis or diffusion [6,8]. Subsequently, dietary AGEs enter circulation, merge with biological AGEs, and are distributed throughout the body, including tissues (such as the gastrointestinal tract, kidneys, liver, heart, lungs and spleen), serum, urine and feces [8]. Accumulated AGEs result in oxidative stress, activate redox-sensitive transcription factors in cells and, ultimately, lead to inflammatory responses or injury [5]. Restricting diet-derived AGEs is a highly effective way to reduce their accumulation in animal and human bodies [5,9]. The accumulation of AGEs in the plasma, kidney and liver increased when mice were fed with a baked chow high-AGEs diet, but these increases normalized after switching to a standard chow diet [9]. Research on animal models also reveals that restricting dietary AGEs intake improves insulin resistance and inhibits the progression of atherosclerosis and renal injury [4].

In addition to AGEs, acrylamide, recognized as a probable carcinogen to humans, is another harmful compound formed by Maillard reactions. Its carcinogenic risks have been reported in rats and mice, yet they remain inconclusive in humans [10]. Acrylamide can be formed in the presence of α-dicarbonyl compounds, such as 3-deoxyglucosone (3-DG) and 2,3-butanedione (2,3-BD), and amino acids, such as asparagine and methionine, at temperatures exceeding 100 °C [11,12,13]. So far, no permissible limits have been set for dietary acrylamide. Nevertheless, because of its potential carcinogenicity, guidance aimed at reducing the acrylamide contents in foods have been issued by regulatory bodies, such as the Food and Drug Administration (FDA) (2016) and the European Union (EU2017/2158) [14].

Previous research has shown that plant-based meat analogues (PBMAs) have higher levels of AGEs compared to most other foods [15,16]. Additionally, the acrylamide contents in commercially sold PBMAs are comparable to categories such as “cake and pastry” and “other products based on cereals” but higher than in “soft breads” [15,17]. Foods in these categories are major contributors to the total acrylamide exposure of adults, elderly and very elderly, with “soft breads” being the second largest contributor to the total acrylamide exposure of toddlers, other children and adolescents [17]. These findings underscore that PBMAs contain significant amounts of AGEs and acrylamide. Recently, PBMAs have attracted increasing attention due to their potential to alleviate protein deficits resulting from population growth, as well as environmental issues associated with intensive livestock farming and animal welfare issues related to slaughter. PBMAs are expected to play a crucial role in future human diets. Therefore, it is essential to gain a thorough understanding of AGEs and acrylamide formation during PBMA production to develop effective mitigation strategies.

Extrusion is one of the most commonly used and commercially applied techniques. During extrusion, proteins undergo mixing, cooking, melting, kneading, shearing, texturizing and shaping, influenced by the combined effects of heating, pressure and shear forces [18]. Concurrently, reactions such as the Maillard reaction and lipid peroxidation occur among proteins and other ingredients. Intermediate compounds of the Maillard reaction, including AGEs and acrylamide, have been found in extruded pet foods, soybean flour and commercial and self-made PBMAs [15,19,20,21]. The extrusion moisture, screw speed, feed rate and barrel temperature are crucial parameters for producing PBMAs with a meat-like fibrous structure [18,22,23]. Changes in these extrusion parameters exert influences on protein unfolding, exposure of reactive sites and re-aggregation, affecting not only the texture of PBMAs but also the occurrence of reactions like the Maillard reaction. Thus, an appropriate configuration of the extrusion parameters should comprehensively consider all of these factors, as well as the physicochemical properties of the raw materials, given their interdependence and coordination.

Despite the development of PBMAs, research has been mainly focused on investigating the formation of fibrous structure, flavor and taste to produce desired meat-like qualities. Even though a small number of studies addressed the presence of hazardous substances such as AGEs and acrylamide in PBMAs [15,19], the impact of extrusion parameters on the formation of these substances have been rarely addressed. Therefore, the current study aimed to investigate the impact of extrusion parameters (including moisture level, screw speed, feed rate and barrel temperature) on the contents of CML, CEL and acrylamide in PBMAs. Additionally, in order to elucidate the underlying mechanisms, the content of protein, amino acids and α-dicarbonyl compounds that are known to be related to the formation of these compounds were also examined. The findings of this study are expected to provide valuable insights into the formation of CML, CEL and acrylamide in PBMAs at different extrusion parameters, therefore serving as a theoretical foundation for developing strategies aiming at reducing their contents.

## 2. Results and Discussion

### 2.1. Moisture, Crude Protein and Amino Acids

Moisture content is a critical parameter during PBMA production. PBMAs produced at moisture levels over 40% are classified as high-moisture products, exhibiting structures and morphological characteristics closely resembling animal-based meat. In this study, the water content in all final products was lower than that of the mixed raw material (Table 1). Increasing the screw speed and the feed rate from 120 to 180 rpm and 4 to 8 kg/h, respectively, led to a slight decrease in PBMAs water content. However, increasing extrusion temperature from 130 to 170 °C, resulted in a significant reduction in water content. The loss of water can be attributed partly to the evaporation of water at high temperature. Another contributing factor may be the conversion of free water to bound water during the extrusion, which potentially enhanced the thermal stability of PBMAs. As the feed rate and temperature increased, the interaction between protein and water intensified, mitigating the conversion of free water to bound water and consequently leading to lower water content in the extrudates [24].

The extrusion process led to a reduction in protein content in PBMAs (Table 1). However, the increase in moisture content impeded this reduction. A decrease in protein in low-moisture extruded samples has been previously reported [25]. Unlike moisture level, variations in the screw speed and the feed rate had no influence on protein content (Table 1). Compared to the raw materials, a lower temperature (130 °C) minimally affected protein content, whereas higher temperatures (150 and 170 °C) caused a decrease in the protein content, indicating protein degradation at higher temperatures. The decrease in protein content during the extrusion process is likely attributed to the formation of volatile compounds. These compounds are generated through protein degradation and subsequently evaporate with water at elevated temperatures [26].

Changes in amino acids in PBMAs extruded under various parameters were shown in Tables S1–S4. With increasing moisture levels, screw speeds, feed rates and barrel temperatures, the total amino acid (TAA) in PBMAs showed similar changing patterns as observed in the protein content (Table 1). Extrusion at lower moisture levels (20 and 30%), a lower feed rate (4 kg/h) and higher barrel temperatures (150 and 170 °C) caused significant decreases in TAA. This can be attributed to accelerated reactions involving amino acids, such as Maillard reactions since these reactions are known to occur more easily under high temperatures and low moisture levels [27]. Additionally, lower feed rates increased the residence time of materials in the extruder barrel, thereby promoting the reaction [18]. During the Maillard reaction, nitrogen-containing volatile compounds such as pyrroles and pyrazines are formed [26]. The increased rate of the Maillard reaction leads to higher levels of these volatile compounds, which subsequently evaporate along with water. This process contributes to a reduction in amino acid content. However, further investigation is required to validate these findings. Changes in proteins and amino acids content in PBMAs indicated that extrusion could influence the nutritional quality of PBMAs. Therefore, when establishing extrusion parameters during the industrial production for PBMAs, consideration should be given not only to achieve desired fibrous structures but also to preserve the nutritional quality.

### 2.2. CML and CEL

**Figure 1:**
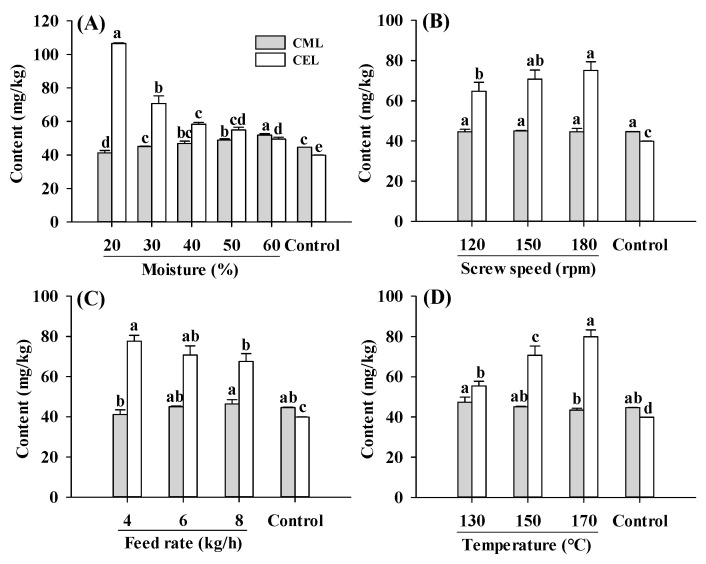
Influence of extrusion moisture level (**A**), screw speed (**B**), feed rate (**C**) and barrel temperature (**D**) on CML and CEL contents of self-made plant-based meat analogues. Values are statistically analyzed by one-way analysis of variance (ANOVA) with Tukey’s HSD test, and expressed as the mean ± standard deviation (SD) (n = 3). Different letters marked on the bars of same compounds indicate statistical differences between values (*p* ≤ 0.05).

Protein-bound CML and CEL contents in PBMAs under different extrusion parameters fell within the range observed in commercially sold and self-made PBMAs (Figure 1) [15,19]. Compared to the raw material (control), extruded samples exhibited a higher total AGE content (the sum of CEL and CML) (Figure 1), indicating that the extrusion process stimulated Maillard reactions. The higher AGE content primarily stemmed from the enhanced formation of CEL, as CEL were significantly higher in all extruded samples than control, whereas the CML content were comparable to control (Figure 1). Compared to the control, changes in screw speeds, feed rates and barrel temperatures led to an increase of 69–94%, 62–88% and 39–101% in CEL, respectively. The enhanced formation of CEL implied that the formation of CEL was more susceptible to extrusion than CML. Higher CEL content in most commercially sold PBMAs, sterilized meat products and sponge cakes were reported previously [15,28,29]. The increase in CEL formation can be attributed to the high barrel temperature applied (≥130 °C), as CEL generation is accelerated at higher temperature (121 °C), whereas CML was favored at milder temperature (85 °C) [29]. Moreover, the reaction of MGO with lysine was at a faster rate compared to the reaction of GO with lysine, leading to greater accumulation of CEL content [28].

The parameters involved in an extrusion process can be categorized into three groups: process parameters (such as moisture, screw speed, feed rate and temperature), system parameters (such as energy input and residence time) and extrudate properties (such as nutritional profile, texture, color and taste) [30]. The process parameters influence the extrudate properties by impacting system parameters such as specific mechanical energy (SME), matrix viscosity and residence time. SME, which represents the work input from the driver motor into the raw material, is related to the breakdown or degradation of material’s molecular structure during extrusion [30].

The CML and CEL contents showed opposite changing patterns in response to the change in moisture level, with CML content slightly increased while CEL decreased with increasing moisture level (Figure 1A). Due to limited studies on CML and CEL levels in extruded PBMAs under different processing parameters, the comparison of present results with other studies was limited. Despite a slight increase in CML content, total AGE content (CML and CEL) was decreased with increasing moisture levels. A decrease in AGEs was also found in pan fried chicken breast [31]. The reduction in AGEs in samples extruded under high moisture levels was attributed to a decrease in matrix viscosity, residence time and SME [30]. The increase in moisture levels firstly decreased the matrix viscosity, then decreased the residence time, and finally reduced the SME [30]. Decreased matrix viscosity resulted in lower maximum shear stress, which further reduced the degree of protein disaggregation and limited the exposure of reactive amino acid groups involved in Maillard reactions, thereby reducing the rate of Maillard reactions [22].

Additionally, an increase in the screw speed from 120 to 180 rpm increased the CEL levels. The higher CEL content in PBMAs produced at high screw speeds may be attributed to the increase in temperature induced by the higher screw speed, which enhanced Maillard reactions [22,23]. Apart from its influence on temperature, an increase in the screw speed could also lead to a shorter residence time of matrix in the extruder barrel and lower maximum shear stress, resulting in fewer disruptions to chemical bond in proteins and less exposure of reactive sites [22]. It was noteworthy that Maillard reactions occurring during the extrusion process are a synergistic effect of the extrusion parameters and matrix. While the Maillard reactions can be promoted by an increase in temperature, they can be alleviated by the decrease in residence time and maximum shear stress. However, despite these factors, AGEs content increased with increasing screw speeds, indicating that the screw speed probably exerts more influence on temperature than on residence time and maximum shear stress. This was in agreement with Emin et al. [22], who reported that thermal stress distribution was more affected by changes in the screw speed than mechanical stress distribution.

With an increase in the feed rate, CML was slightly increased, whereas CEL was decreased. Moreover, the total AGEs content decreased slightly, possibly due to the decrease in residence time caused by the increased feed rate [32]. As expected, CEL content significantly increased with an increase in barrel temperature from 130 °C to 170 °C. Although CML content slightly decreased, the total AGEs content increased, consistent with previous research, which reported that Maillard reactions were enhanced by high temperature [27]. In addition, temperature can also result in lower matrix viscosity, SME and disaggregation degree of proteins, which could reduce the rate of Maillard reactions [22]. In the present study, the levels of AGEs increased with rising temperatures, indicating that the impact of temperature on the Maillard reactions was more significant compared to its effect on matrix viscosity.

Variations in the extrusion parameters resulted in different changing patterns for the contents of CML and CEL. This was inconsistent with previous findings that CML and CEL have similar changing patterns under same process or storage conditions [3,19]. It was speculated that the differences stem from variations in formation rather than degradation of CML and CEL, given their stability. Specifically, CML primarily forms by the action of GO with Lys or Arg residues, while CEL forms by the reaction of MGO with Lys residues. Therefore, subsequent sections will assess GO and MGO contents and their correlations with CML and CEL.

Protein-bound AGEs can be digested into smaller peptide- or protein-bound AGEs, which are subsequently absorbed by intestinal tract and accumulate in the human body. Absorbed AGEs can bind to tissue proteins by covalent bonds, promoting protein aggregation or tissue stiffness, and eventually altering their original functions [9]. In addition to their direct role in inducing inflammatory responses or injury, glycation reduces the digestibility of glycated proteins, thereby diminishing the nutritional quality of foods [6,33]. Moreover, the amount of absorbed AGEs is directly proportional with the amount ingested [7]. Therefore, reducing AGE contents in PBMAs is crucial for both health and nutritional purposes. Additionally, extrusion parameters affect the Maillard reactions by altering system parameters such as energy input, viscosity and residence time. According to the results, it is speculated that higher moisture levels alleviated the accumulation of AGEs by decreasing matrix viscosity, residence time and SME, while higher feed rates reduced AGEs formation by shortening the residence time. Conversely, higher screw speeds aggregated the accumulation of AGEs by increasing the temperature. Therefore, in order to reduce AGEs contents in PBMAs, it is necessary to increase moisture and feed rates, while decreasing the screw speed and barrel temperature. This aligns with a previous report that Maillard reactions are favored by high temperatures, high shear and low moisture [27].

### 2.3. Acrylamide

**Figure 2:**
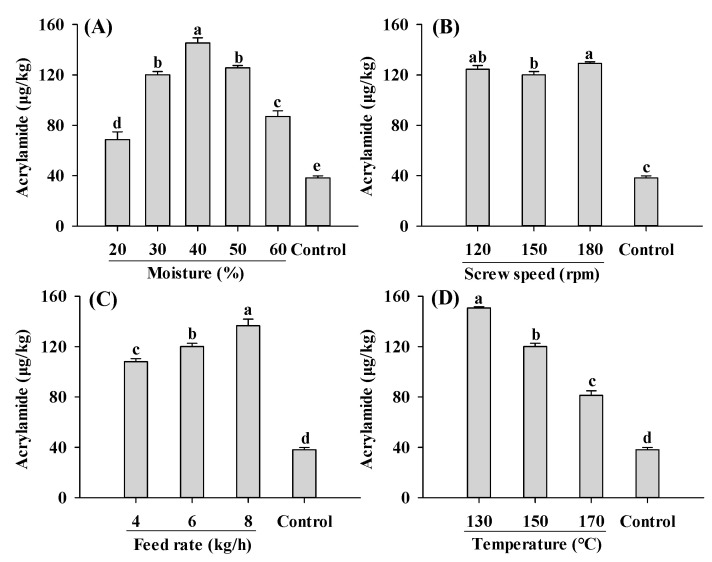
Influence of extrusion moisture (**A**), screw speed (**B**), feed rate (**C**) and temperature (**D**) on acrylamide content of self-made plant-based meat analogues. Values are statistically analyzed by one-way analysis of variance (ANOVA) with Tukey’s HSD test, and expressed as the mean ± standard deviation (SD) (n = 3). Different letters marked on the bars indicate statistical differences between values (*p* ≤ 0.05).

Compared to the control, acrylamide contents in all extruded samples were significantly increased (Figure 2). An increase in acrylamide content in extruded soybean kernels was reported previously [34]. The lowest and highest acrylamide content were found in PBMAs extruded by 20% moisture and 130 °C, respectively. These values fall within the range reported for commercially sold PBMAs (32–187 μg/kg) [15]. Acrylamide contents in PBMAs showed a bell-shaped pattern with increasing moisture level, with the highest acrylamide content at 40% moisture content (Figure 2A). These results indicated that for low-moisture extrusion (≤40%), increasing moisture level promoted acrylamide formation, whereas for high-moisture extrusion, increasing moisture level inhibited its formation. However, Mulla et al. [35] reported a decrease in acrylamide in low-moisture extruded samples with increasing moisture. The controversial results were likely attributed to differences in the extrusion materials used. In the present study, soy protein isolate and corn starch were used, whereas potato flour and semolina were used in the study of Mulla et al. [35]. This discrepancy has been supported by a previous study demonstrating that acrylamide levels in samples extruded using different materials exhibited opposite changing patterns as moisture levels increased from 12% to 14% [36]. The decrease in acrylamide content in higher moisture extruded samples may also result from the decrease in product temperature, which reduced acrylamide formation [23]. Moreover, increased moisture levels may lead to decreased matrix viscosity and SME, which could inhibit Maillard reactions and consequently reduce acrylamide formation [22,30].

Changes in the screw speed only slightly influenced acrylamide (Figure 2B). However, decreased acrylamide content with an increase in the screw speed was reported by Mulla [35]. Acrylamide content increased with increasing feed rates and decreasing barrel temperature, with the highest amount observed at 8 kg/h and 130 °C, respectively (Figure 2C,D). A decrease in acrylamide content at higher temperatures was also reported by Mottram et al. [11]. The decrease was probably due to the degradation of acrylamide as well as the Micheal addition reaction between acrylamide and 3-aminopropanamide (3-APA) [37]. During food production, 3-APA, which was considered the direct precursor to acrylamide, can form through the reaction between Asp and reducing sugars [38]. The formation and reduction of acrylamide caused by 3-APA occur simultaneously during the thermal treatment. Moreover, within the range of 120–160 °C, the degradation of acrylamide and the reduction of acrylamide in reaction systems of acrylamide and 3-APA increases with temperature [37]. The current findings suggest that as extrusion temperature increases, the rate of acrylamide elimination accelerates more rapidly than its formation rate. This observation aligns with the findings of de Vleeschouwer et al. [39]. However, Žilić et al. [34] found that acrylamide in the extruded soybean kernels increased as temperature increased from 110 °C to 140 °C. Similar patterns were also reported by Mulla et al. [35] in extruded potato flour and semolina blends. Further systematic studies are needed to explore the relationship between acrylamide and 3-APA during the extrusion. In general, maintaining very low or very high moisture levels, along with high feed rates and temperatures, proves beneficial for producing PBMAs with low acrylamide content.

The formation of acrylamide during the extrusion process is a complex chemical phenomenon. For example, amino acids generated from protein degradation during extrusion play a crucial role in acrylamide formation. Gly, Asp and Cys have been shown to inhibit acrylamide formation in model systems, whereas Val, Ala and glutamine promote its formation [40]. Additionally, competition between Lys and Asn for glucose, GO and MGO also influences acrylamide, as well as CML and CEL formation [41]. Different from amino acids, the type of sugars has little influence on the formation of acrylamide [12]. α-Dicarbonyl compounds such as MGO, GO and 2,3-BD are precursors to acrylamide in the Maillard reactions [11,13,40]. In this study, the influence of moisture on acrylamide formation was likely due to its influence on these α-dicarbonyl compounds. Therefore, investigating the formation of α-dicarbonyl compounds during the extrusion process is important to understanding acrylamide and AGEs formations.

### 2.4. α-Dicarbonyl Compounds

**Figure 3:**
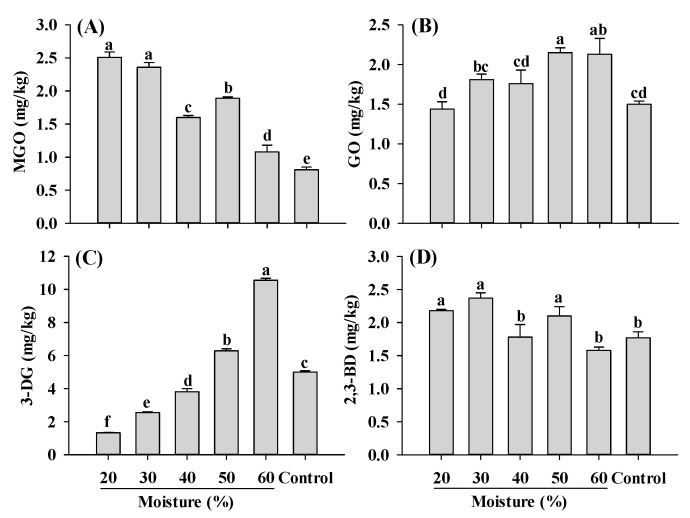
Influence of extrusion moisture on MGO (**A**), GO (**B**), 3-DG (**C**) and 2,3-BD (**D**) content of self-made plant-based meat analogues. Values are statistically analyzed by one-way analysis of variance (ANOVA) with Tukey’s HSD test, and expressed as the mean ± standard deviation (SD) (n = 3). Different letters marked on the bars indicate statistical differences between values (*p* ≤ 0.05). MGO: methylglyoxal; GO: glyoxal; 3-DG: deoxyglucosone; 2,3-BD: 2,3-butanedione.

MGO, GO, 3-DG and 2,3-BD are important and widely studied α-dicarbonyl compounds, due to their involvement in the formation of harmful compounds AGEs and acrylamide [2,11]. Carbohydrates play a crucial role in the formation of the fibrous structure of PBMAs and are widely used in its production. In this study, corn starch was added for the extrusion. During the extrusion of starch, significant amounts of reducing sugars were formed, ranging from 750 to 850 mg/100 g depending on the extrusion temperature and moisture conditions [42]. These reducing sugars such as glucose, undergo reactions such as dehydration and cleavage, leading to the formation of 3-DG and 1-deoxyglucosone (1-DG), which further break down to form MGO [43,44,45]. GO can be generated through retro-aldol scission of glucose and the degradation of fructoselysine [45]. The contents of MGO, GO, 3-DG and 2,3-BD in PBMAs of present study were comparable to those found in baked bread, dried apple, sesame seeds and dried dates, respectively [46]. Compared to control, MGO content significantly increased in samples extruded at 20–60% moisture (Figure 3). Different from MGO, GO in extruded samples only increased slightly with increasing moisture level. The changing patterns of MGO and GO, which were the precursors of CEL and CML, were consistent with CEL and CML along with the moisture content (Figure 1). The most significant influence of moisture content on α-dicarbonyl compounds observed was 3-DG (Figure 3C). The increase in 3-DG with increasing moisture levels indicated that high moisture content facilitated its formation. Compared to the control, low-moisture extrusion (≤40%) decreased the content of 3-DG, whereas high-moisture extrusion (>40%) increased its content. Regarding 2,3-BD, which is also involved in acrylamide formation, mixed pattern was observed. Samples extruded at 40 and 60% moisture exhibited lower 2,3-BD levels compared to the control; however, other samples showed higher 2,3-BD content than the control (Figure 3D). To the best of our knowledge, this is the first study investigating the changes in α-dicarbonyl compounds during the extrusion process; therefore, it was impossible to compare the current findings with previous studies. Nonetheless, the results indicate that high moisture extrusion promotes the formation of GO and 3-DG, while alleviating the formation of MGO and 2,3-BD.

**Figure 4:**
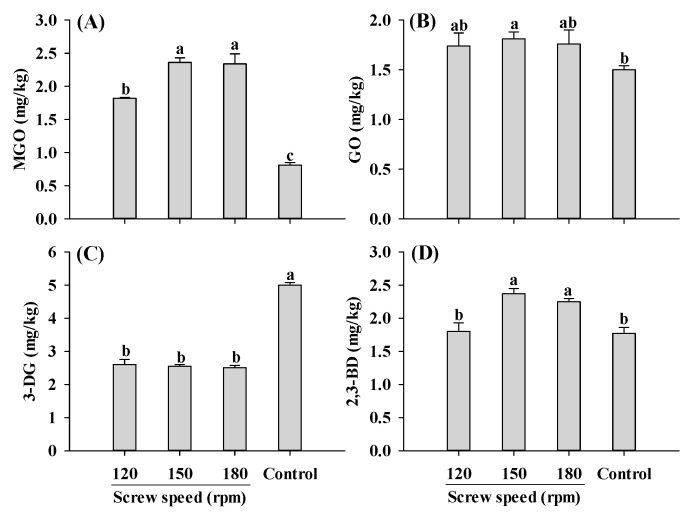
Influence of the screw speed on MGO (**A**), GO (**B**), 3-DG (**C**) and 2,3-BD (**D**) content of self-made plant-based meat analogues. Values are statistically analyzed by one-way analysis of variance (ANOVA) with Tukey’s HSD test, and expressed as the mean ± standard deviation (SD) (n = 3). Different letters marked on the bars indicate statistical differences between values (*p* ≤ 0.05). MGO: methylglyoxal; GO: glyoxal; 3-DG: deoxyglucosone; 2,3-BD: 2,3-butanedione.

Furthermore, MGO content in the extruded samples was higher than control, with the lowest MGO content observed at a screw speed of 120 rpm (Figure 4A). In the screw speed range of 120–180 rpm, the levels of GO and 3-DG were not influenced by the changes in the screw speed (Figure 4B,C). Consistently, the changing patterns of GO, the precursor to CML, were similar to those observed for CML (Figure 1B and Figure 4B). As for 2,3-BD, its content increased at screw speeds of 150 and 180 rpm, but was not significantly altered at 120 rpm. The increase in MGO and 2,3-BD contents with increasing screw speeds was likely due to the increase in material temperature, which accelerated the reactions. It was previously reported that increasing screw speeds result in a higher material temperature, which could promote protein unfolding, chemical bonds disruption and reactive sites exposure [18,22,23].

**Figure 5:**
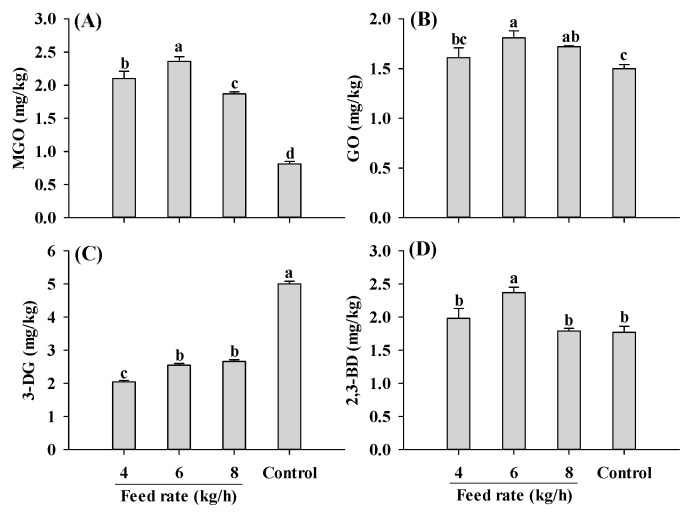
Influence of the feed rate on MGO (**A**), GO (**B**), 3-DG (**C**) and 2,3-BD (**D**) content of self-made plant-based meat analogues. Values are statistically analyzed by one-way analysis of variance (ANOVA) with Tukey’s HSD test, and expressed as the mean ± standard deviation (SD) (n = 3). Different letters marked on the bars indicate statistical differences between values (*p* ≤ 0.05). MGO: methylglyoxal; GO: glyoxal; 3-DG: deoxyglucosone; 2,3-BD: 2,3-butanedione.

Regardless of the feed rate applied, samples extruded at a 30% moisture level had higher MGO and lower 3-DG than control, consistent with the results in Figure 3A,B (Figure 5). An increase in the feed rate initially led to a rise and then a decline in MGO content. A similar fluctuating pattern was also found in GO and 2,3-BD contents. The changing patterns observed for the α-dicarbonyl compounds was probably attributed to the increase in melt viscosity at low feed rates, which led to higher shear stress and proteins disaggregation, collectively accelerating Maillard reactions [22,47]. However, with a continuous increase in the feed rate, more materials were fed into the extruder, causing a continuous rise in melt viscosity. This higher melt viscosity resulted in incomplete denaturation of the proteins [47]. Further investigation is required to verify the speculations.

**Figure 6:**
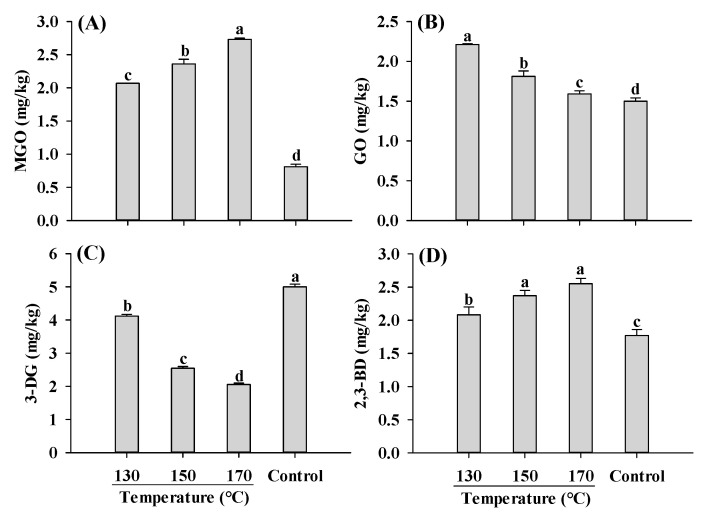
Influence of barrel temperature on MGO (**A**), GO (**B**), 3-DG (**C**) and 2,3-BD (**D**) content of self-made plant-based meat analogues. Values are statistically analyzed by one-way analysis of variance (ANOVA) with Tukey’s HSD test, and expressed as the mean ± standard deviation (SD) (n = 3). Different letters marked on the bars indicate statistical differences between values (*p* ≤ 0.05). MGO: methylglyoxal; GO: glyoxal; 3-DG: deoxyglucosone; 2,3-BD: 2,3-butanedione.

Similar to AGEs and acrylamide, the content of α-dicarbonyl compounds was significantly affected by temperature. Compared to the control, extruded samples exhibited higher levels of MGO, GO and 2,3-BD, but lower levels of 3-DG (Figure 6). As the temperature increased, the concentrations of MGO and 2,3-BD were increased, whereas GO and 3-DG were decreased. The decrease in 3-DG with increasing temperature has been reported in the model systems and roasted sesame seeds [44,45]. The decrease in 3-DG and GO, which are products of glucose degradation, was probably due to the decreased rate of glucose degradation at higher temperatures. Despite the decrease in 3-DG content with increasing temperature, the content of MGO, a degradation product of 3-DG, increased. This could be attributed to the fact that MGO is predominantly formed through the degradation of 1-DG rather than 3-DG [45]. Another α-dicarbonyl compound, 2,3-BD, which is produced through the degradation of 1-DG, also increased with increasing temperatures, consistent with the findings in glucose model systems [44].

When extrusion parameters were varied, MGO and GO, precursors of CEL and CME, respectively, exhibited similar changing patterns as CEL and CML (Figure 1D and Figure 6A,B). This finding supports our hypothesis that extrusion parameters influence the contents of AGEs by affecting their formations from α-dicarbonyl compounds. Variations in moisture content, screw speed, feed rate and temperature impact the matrix viscosity, residence time and temperature, which in turn influence the formation of α-dicarbonyl compounds and consequently affect AGEs formation. α-Dicarbonyl compounds in foods are associated with the formation of dietary AGEs, while ingested α-dicarbonyl compounds contribute to the formation of endogenous AGEs [6,9]. Therefore, optimizing extrusion parameters to reduce the content of α-dicarbonyl compounds in PBMAs not only decreases the AGEs formation in PBMAs, but also mitigates the formation of endogenous AGEs. Further systematic studies are needed to better understand the formation and degradation of α-dicarbonyl compounds under varying extrusion parameters.

### 2.5. Correlation Analysis

**Figure 7:**
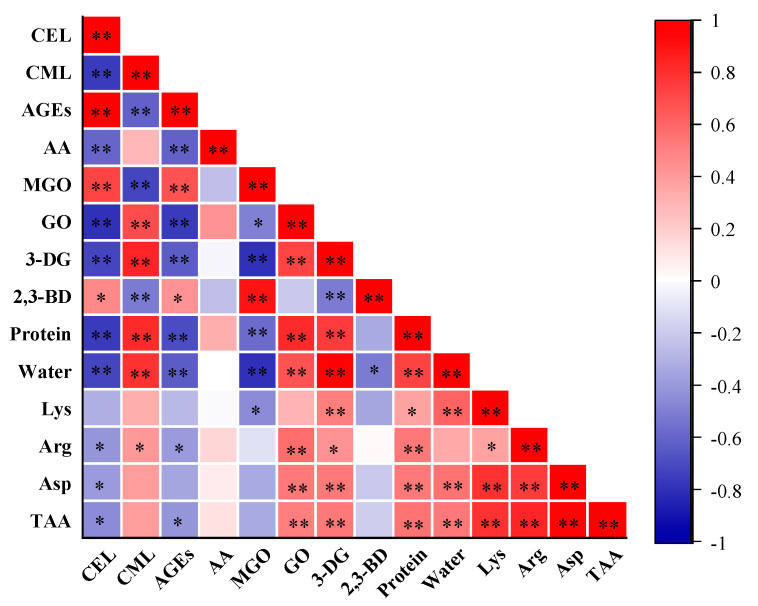
Correlations of Maillard reactive compounds with α-dicarbonyl compounds (MGO, GO, 3-DG and 2,3-BD), protein, water and amino acids in self-made plant-based meat analogues produced at various extrusion parameters (n = 33). Correlation analysis is performed using two-tailed Pearson’s correlation. *p*-Values were adjusted for multiple comparisons using the false discovery rate (FDR). *, adjusted *p* value < 0.05, compounds were significantly correlated at 0.05 level; **, adjusted *p* value < 0.01, compounds were significantly correlated at 0.01 level. AA: acrylamide; AGEs: the sum of CML and CEL; MGO: methylglyoxal; GO: glyoxal; 3-DG: deoxyglucosone; 2,3-BD: 2,3-butanedione; TAA: total amino acids.

CML exhibited a positive correlation (FDR-adjusted *p* < 0.01, r = 0.80) with water content in PBMAs, while CEL exhibited a negative correlation (FDR-adjusted *p* < 0.01, r = −0.71) (Figure 7). These changing patterns were in line with the observed effects of increasing moisture content (Figure 2A). The total AGEs content also exhibited a negative correlation with water content (FDR-adjusted *p* < 0.01, r = −0.63). Consistently, a negative correlation between moisture and AGEs has been reported in pan-fried chicken breast [31]. These results indicated that PBMAs with higher water content tend to have lower CEL and total AGEs contents. These results suggest that extrusion methods that can maintain the water content of PBMAs may help mitigate the accumulation of CEL and total AGEs contents.

Although Lys is recognized as a key reactant in the Maillard reactions for AGEs formation, no significant correlation was found between Lys and CML, CEL, or total AGEs contents (Figure 7). Arg is reported to react with GO to form CML [3]. Correlation analysis revealed a positive relationship between Arg content in PBMAs and CML (Figure 7). Correlations between CML, CEL and other amino acids were presented in Table S5. Although Asp is reported to be a crucial participant in acrylamide formation [11], no significant correlation was observed between Asp and acrylamide content (Figure 7). This was likely due to the determination of total Aps rather than free Aps.

MGO and GO are precursors of CEL and CML, respectively. As expected, a positive correlation was observed between MGO and CEL (FDR-adjusted *p* < 0.01, r = 0.73), and between GO and CML (FDR-adjusted *p* < 0.01, r = 0.68). These results further validate that extrusion parameters impact the formation of CML and CEL by influencing the formation of GO and MGO. However, despite 3-DG and 2,3-BD being involved in the formation of acrylamide [11,12,13], no significant correlation was observed between these compounds. Interestingly, MGO content in PBMAs showed a negative correlation with protein and water content, while GO and 3-DG displayed a positive correlation (Figure 7). Consistent with their precursors, MGO and GO, CEL content negatively correlated with protein and water content, whereas CML showed a positive correlation (Figure 7). Additionally, the total AGEs content was negatively correlated with protein and water content. These results indicate that an increase in protein content can accelerate the CML formation in PBMAs, while it tends to reduce CEL formation. However, it has been observed that protein content in beef or pork had very little impact on the formation of protein-bound AGEs during sterilization, whereas protein content in pan-fried chicken breast is positively correlated with AGEs [31,48]. This discrepancy is likely attributed to the complexity of the food matrix and the multiple reactions involved in AGEs formations. More systematic investigations are needed to elucidate the underlying mechanisms.

## 3. Materials and Methods

### 3.1. Materials

Soy protein isolate and corn starch were purchased from Linyi Shansong Biological Products Co., Ltd. (Linyi, China) and Jideli Food Co., Ltd. (Beijing, China), respectively. CML, CEL, CML-d4 and CEL-d4 with a purity of 98% were obtained from Toronto Research Chemicals Inc. (TRC, Toronto, ON, Canada). Acylamide-2,3,3-d3 with a purity of 98% was obtained from Shanghai Macklin Biochemical Co., Ltd. (Shanghai, China). Methylglyoxal (MGO, 40 wt.% in water), glyoxal (GO, 40 wt.% in water), 3-deoxyglucoronone (3-DG, 75%), 2,3-butanedione (2,3-BD), o-phenylenediamine (OPD), diethylenetriaminepentaacetic acid (DETAPAC) and acrylamide with a purity of 99% were obtained from Merck KGaA (Darmstadt, Germany). Amino acids standard solution was purchased from Waters Corporation (Waters, Milform, MA, USA).

### 3.2. Extrusion

Soy protein isolate and corn starch at a ratio of 9:1 were used for extrusion. Extrusion was carried out using a pilot-scale twin-screw extruder (LS-30, Jinan Sinopuff Machinery Co., Ltd., Jinan, China), featuring a 35 mm screw diameter and a screw length/diameter ratio of 40:1. The extruder barrel comprises a feeding zone and 10 heating zones. During extrusion, the temperature of the first 5 heating zones from the feed to the die was maintained at 80, 80, 85, 90 and 100 °C, respectively, while the temperatures of the last 5 heating zones was set at 120, 140, 150, 150 and 150 °C. The screw speed was 150 rpm, with a feed rate of 6.0 kg/h for raw material and 2.57 kg/h for water. The moisture content of the mixed raw material was 30%. For single-factor experiments, the relevant factors were varied while keeping the other parameters constant. In the temperature experiment, the temperature of the last 5 heating zones was set as follows: (1) 110, 120, 130, 130 and 130 °C; (2) 120, 140, 150, 150 and 150 °C; (3) 120, 140, 170, 170 and 170 °C. In the screw speed experiment, screw speeds of 120, 150 and 180 rpm were tested. In the feed rate experiment, feed rates of 4.0, 6.0 and 8.0 kg/h were used. In the moisture content experiment, moisture contents of 20, 30, 40, 50 and 60% were used. The range of each parameter variation was determined based on preliminary experiments. The criterion for selection was to achieve the formation of extrudates with a fibrous structure when using soy protein isolate/corn starch (9:1, *w*/*w*) as the extrusion material. The moisture of the mixed material was obtained by continuously injecting water into the extruder through an inlet port at rates of 1.5, 2.57, 4.0, 6.0 and 9.0 kg/h, respectively. Extruded samples were collected once steady-state extrusion was achieved (within 5 min). Samples were then cooled to 25 °C, diced, freeze-dried using a vacuum freeze drier (Scientz-10N, Ningbo Scientz Biotechnology Co., Ltd., Ningbo, China), ground into powders using a grinder (SD-JR05, Sande Electric Appliance Co., Ltd., Foshan, China), and sieved with a 60-mesh sieve. The fine powders were stored at −80 °C until further analysis.

### 3.3. Crude Protein, Moisture and Amino Acids Contents

Crude protein content was determined by a Dumas azotometer (D200, Hanon Advanced Technology Group Co., Jinan, China). Moisture content was calculated by the weight loss of 1.0 g sample in a weighing bottle before and after drying in an oven at 105 °C for 24 h. Amino acids were extracted and detected according to Deng et al. [49] with slight modifications. For amino acid extraction, excluding Trp, a 50.00 mg sample was mixed with 8 mL of HCl (6 mol/L) and hydrolyzed at 110 °C for 24 h. After cooling to room temperature (approximately 25 °C), the hydrolysate was filtered through a 0.22 μm filter. Then, 0.1 mL was dried by a termovap sample concentrator, redissolved in 1.0 mL of 0.1% formic acid solution, and used for the analysis of amino acids. For Trp extraction, a 50.00 mg sample was mixed with 5 mL of lithium hydroxide solution (5 mol/L) and hydrolyzed at 110 °C for 24 h. After cooling to room temperature, the pH of the hydrolysate was adjusted to 6–8. After filtration, the volume was made to 25 mL with distilled water. The solution was used for Trp determination.

Amino acid analysis was performed on a Waters ACQUITY UPLC H-Class chromatographic system (Waters, Milform, MA, USA) coupled with a XEVO TQ-XS tandem triple quadrupole mass spectrometer (Waters, Milform, MA, USA) (UPLC-QqQ-MS/MS). Separation was conducted by a Waters ACQUITY HSS T3 column (1.8 μm, 2.1 mm × 150 mm), with a column temperature set at 35 °C and an injection volume of 2 μL. Ultrapure water with 0.1% formic acid (A) and acetonitrile with 0.1% formic acid (B) were used as mobile phase and the flow rate was 0.2 mL/min. The gradient elution program was as follows: 0 min, 100% A; 3 min, 100% A, curve 6; 6 min, 90% A, curve 6; 10 min, 65% A, curve 6; 11 min, 5% A, curve 6; 13.5 min, 5% A, curve 1; 18 min, 100% A, curve 1.

Detection of amino acid parent and fragment ions was performed in positive electrospray ionization (ESI+) with multiple reaction monitoring (MRM) mode. The ESI conditions were set as follows: desolvation temperature, 550 °C; ion source temperature, 150 °C; capillary voltage, 3.0 kV; cone gas flow rate, 150 L/h; and collision gas flow rate, 0.16 mL/min; desolvation gas flow rate, 1000 L/h. Retention time, precursor ion, product ion, collision energy and cone voltage of amino acids were summarized in Table S6. Standard solutions were prepared at the concentration of 0.01 to 10 μmol/L. Results were expressed as mg/g. The UHPLC-MS/MS chromatograms of amino acids were presented in Figure S1.

### 3.4. CML and CEL

Extraction, detection and separation of protein-bound CML and CEL in PBMAs were performed according to the method reported by Fu et al. [15], with minor modification utilizing a Waters ACQUITY HSS T3 column (1.8 μm, 2.1 mm × 150 mm). The retention time, precursor ion, product ion, collision energy and cone voltage for CML, CEL as well as their isotope-labeled internal standards (CML-d_4_ and CEL-d_4_) were summarized in Table S7. The UHPLC-MS/MS chromatograms of CML, CEL and their internal standards were presented in Figure S2.

### 3.5. Acrylamide

Methods used for the extraction and separation of acrylamide were the same as Fu et al. [15]. Acrylamide quantification was performed on a UPLC-QqQ-MS/MS. The retention time, precursor ion, product ion, collision energy and cone voltage of acrylamide and its isotope-labeled internal standard (acrylamide-d_3_) were summarized in Table S7. The UHPLC-MS/MS chromatograms of acrylamide and acrylamide-d3 were presented in Figure S3.

### 3.6. α-Dicarbonyl Compounds

α-Dicarbonyl compounds (MGO, GO, 3-DG and 2,3-BD) in extruded samples were quantified following the methods of Degen et al. [2] and Deng et al. [49], with slight modifications. A sample of 0.5 g was weighted into a 50 mL centrifuge tube, to which 5 mL of distilled water was added. After vigorous mixing for 3 min, the mixture was extracted at room temperature for 1 h. Then, 5 mL of acetonitrile was added, and the mixture was incubated at −20 °C for 1 h. After centrifuging at 10,000× *g* for 10 min, 0.15 mL of sodium phosphate buffer (0.5 mom/L, pH 7) was added into 0.5 mL of supernatant solution, and the mixture was derivatized by 0.15 mL OPD (0.2%, *w*/*v*, containing 11 mmol/L DETAPAC). The mixture was kept in dark for 14 h, after which the mixture was filtered through a 0.22 μm membrane filter for determination. Mixed standard solutions were prepared at the concentration of 0–0.5 mg/L and derivatized by OPD as samples. To exclude the interference caused by sample, a blank of each sample without the addition of OPD was also prepared.

The identification and quantification of derivatives of α-dicarbonyl compound were conducted using an UPLC-QqQ-MS/MS equipped with a Waters ACQUITY HSS T3 column (1.8 μm, 2.1 mm × 150 mm). The column temperature was 30 °C and the injection volume was 1 μL. Acetonitrile (A) and 0.1% formic acid solution (B) were used as mobile phases and set as follows: 0 min, 2% A; 1.2 min, 10% A; 3.5 min, 30% A; 5.0 min, 45% A; 7.5 min, 90% A; 8.0 min, 2% A; 11.0 min, 2% A. The flow rate was 0.3 mL/min. Detection of parent and fragment ions of quinoxalines were performed in ESI+ with MRM mode. ESI conditions were optimized as follows: capillary voltage, 3 kV; ion source temperature, 120 °C; desolvation temperature, 450 °C; desolvation gas flow rate, 1000 L/h; collision gas flow rate, 0.17 mL/min; cone gas flow rate, 150 L/h. Retention time, precursor ion, product ion, collision energy and cone voltage of α-dicarbonyl compounds were summarized in Table S7. Result was expressed as mg/kg. The UHPLC-MS/MS chromatograms of α-dicarbonyl compounds were presented in Figure S4.

### 3.7. Statistical Analysis

All determinations were conducted in triplicate. Data were expressed as the means ± standard deviations (SD) and were subjected to one-way analysis of variance (ANOVA) using SPSS 20.0 (IBM SPSS statistic software, v. 20.0, SPSS Inc., Chicago, IL, USA). Statistical differences among groups were determined using Tukey’s HSD test (*p* ≤ 0.05). Two-tailed Pearson’s correlation analysis was performed using OriginPro 2021 (OriginLab Corporation, Northampton, MA, USA) and SPSS 20.0. *p*-Values were adjusted for multiple comparisons using the Benjamini and Hochberg false discovery rate (FDR) method. A FDR-adjusted *p* value of less than 0.05 was considered as a statistically significant correlation.

## 4. Conclusions

The extrusion process significantly enhanced the accumulation of CML, CEL, acrylamide and MGO in PBMAs. Moisture and temperature were the predominant factors influencing the formation of CML, CEL, acrylamide and α-dicarbonyl compounds. CML and CEL showed similar changing patterns to extrusion parameters as their precursors GO and MGO. Except for PBMAs extruded under 60% moisture content, PBMAs extruded under various parameters all exhibited higher CEL content than CML. With increasing moisture levels, CEL content decreased, whereas CML increased in PBMAs. At low moisture levels (≤40%), acrylamide content in the extrudate increased with increasing moisture content, while at high moisture levels (>40%), it decreased along with the increase in moisture content. Increased temperatures promoted the accumulation of CEL, MGO and 2,3-BD, and at the same time reduced the accumulation of CML, acrylamide, GO and 3-DG. Correlation analysis showed that CEL, MGO and total AGEs content were negatively correlated with protein and water content, whereas CML, GO and 3-DG were positively correlated with these levels. CEL was negatively correlated with Arg, Asp and TAA, whereas positive correlation was found between CML and Arg. It was speculated that variations in extrusion parameters impact the formation and degradation of α-dicarbonyl compounds by affecting matrix viscosity, residence time in barrel, shear stress and barrel temperature. These factors subsequently influence the accumulation of AGEs and acrylamide in PBMAs. In summary, PBMAs extruded under higher moisture and feed rates, lower screw speeds and barrel temperatures exhibited lower CEL and total AGEs content, while PBMA extruded under lower or higher moisture, lower feed rates and higher barrel temperatures had lower acrylamide content. The findings of this study are significant to understanding the formation of AGEs and acrylamide and drawing attention to their presence in PBMAs. Furthermore, these results can serve as guidelines to reducing the levels of AGEs and acrylamide in PBMAs. Future research will focus on exploring methods to mitigate the content of harmful compounds in PBMAs.

## Figures and Tables

**Table 1:** Effect of extrusion parameters on water, protein and total amino acid contents of plant-based meat analogues.

Parameter		Content		
		Water (%)	Protein (%)	TAA (mg/g)
Moisture (%)	20	15.5 ± 0.4 ^e^	79.2 ± 0.5 ^c^	678 ± 3 ^b^
	30	21.5 ± 0.5 ^d^	80.3 ± 0.3 ^c^	679 ± 7 ^b^
	40	35.1 ± 0.5 ^c^	81.8 ± 1.1 ^b^	701 ± 12 ^a^
	50	44.3 ± 0.8 ^b^	82.8 ± 0.4 ^ab^	704 ± 6 ^a^
	60	56.3 ± 0.1 ^a^	83.5 ± 0.8 ^ab^	712 ± 3 ^a^
	Raw material	6.8 ± 0.1 ^f^	84.1 ± 0.4 ^a^	702 ± 7 ^a^
Screw speed (rpm)	120	21.1 ± 0.1 ^a^	80.4 ± 1 ^b^	686 ± 10 ^a^
	150	21.5 ± 0.5 ^a^	80.3 ± 0.3 ^b^	679 ± 7 ^a^
	180	20.4 ± 0.1 ^b^	80.4 ± 0.8 ^b^	687 ± 13 ^a^
	Raw material	6.8 ± 0.1 ^c^	84.1 ± 0.4 ^a^	701 ± 7 ^a^
Feed rate (kg/h)	4	21.3 ± 0.2 ^ab^	79.7 ± 0.9 ^b^	646 ± 37 ^b^
	6	21.5 ± 0.5 ^a^	80.3 ± 0.3 ^b^	679 ± 7 ^ab^
	8	20.8 ± 0.3 ^b^	80.8 ± 0.2 ^b^	684 ± 6 ^ab^
	Raw material	6.8 ± 0.1 ^c^	84.1 ± 0.4 ^a^	701 ± 7 ^a^
Barrel	130	25.0 ± 0.4 ^a^	83.7 ± 0.3 ^a^	715 ± 6 ^a^
temperature (°C)	150	21.5 ± 0.5 ^b^	80.3 ± 0.3 ^b^	679 ± 7 ^b^
	170	19.8 ± 0.5 ^c^	80.7 ± 0.4 ^b^	687 ± 13 ^b^
	Raw material	6.8 ± 0.1 ^d^	84.1 ± 0.4 ^a^	701 ± 7 ^a^

Values are statistically analyzed by one-way analysis of variance (ANOVA) with Tukey’s HSD test, and presented as the means ± SD (n = 3). Different superscript letters marked on the values in the column of per parameter indicate significant differences (*p* ≤ 0.05).

## Data Availability

Data will be made available on request.

## Supplementary Materials

The following supporting information can be downloaded at: https://www.mdpi.com/article/10.3390/ijms25168668/s1.
