# Supplementary material for: Impact of Extrusion Parameters on the Formation of Nε-(Carboxymethyl)lysine, Nε-(Carboxyethyl)lysine and Acrylamide in Plant-Based Meat Analogues
Source: Int J Mol Sci. 2024 Aug 8;25(16):8668. doi: 10.3390/ijms25168668 (PMC11354377; doi:10.3390/ijms25168668)
Supplement: Supplementary file 1 [file ijms-25-08668-s001.zip › ijms-3098942-supplementary.pdf]

Supporting Information

Impact of Extrusion Parameters on the Formation of *N*$^{\varepsilon}$-(Carboxymethyl)lysine, *N*$^{\varepsilon}$-(Carboxyethyl)lysine and Acrylamide in Plant-Based Meat Analogues

Yurong Ma [1,2,3], Shuang Fu [1,2,3], Ka-Wing Cheng [1,2,3] and Bin Liu [1,2,3,*]

[1] College of Chemistry and Environmental Engineering, Shenzhen University, Shenzhen 518060, China; yurongm@163.com (Y.M.); fushuang0322@163.com (S.F.); kwcheng@szu.edu.cn (K.-W.C.)
[2] Shenzhen Key Laboratory of Food Nutrition and Health, Shenzhen University, Shenzhen 518060, China
[3] Institute for Innovative Development of Food Industry, Shenzhen University, Shenzhen 518060, China
* Correspondence: liubin@szu.edu.cn

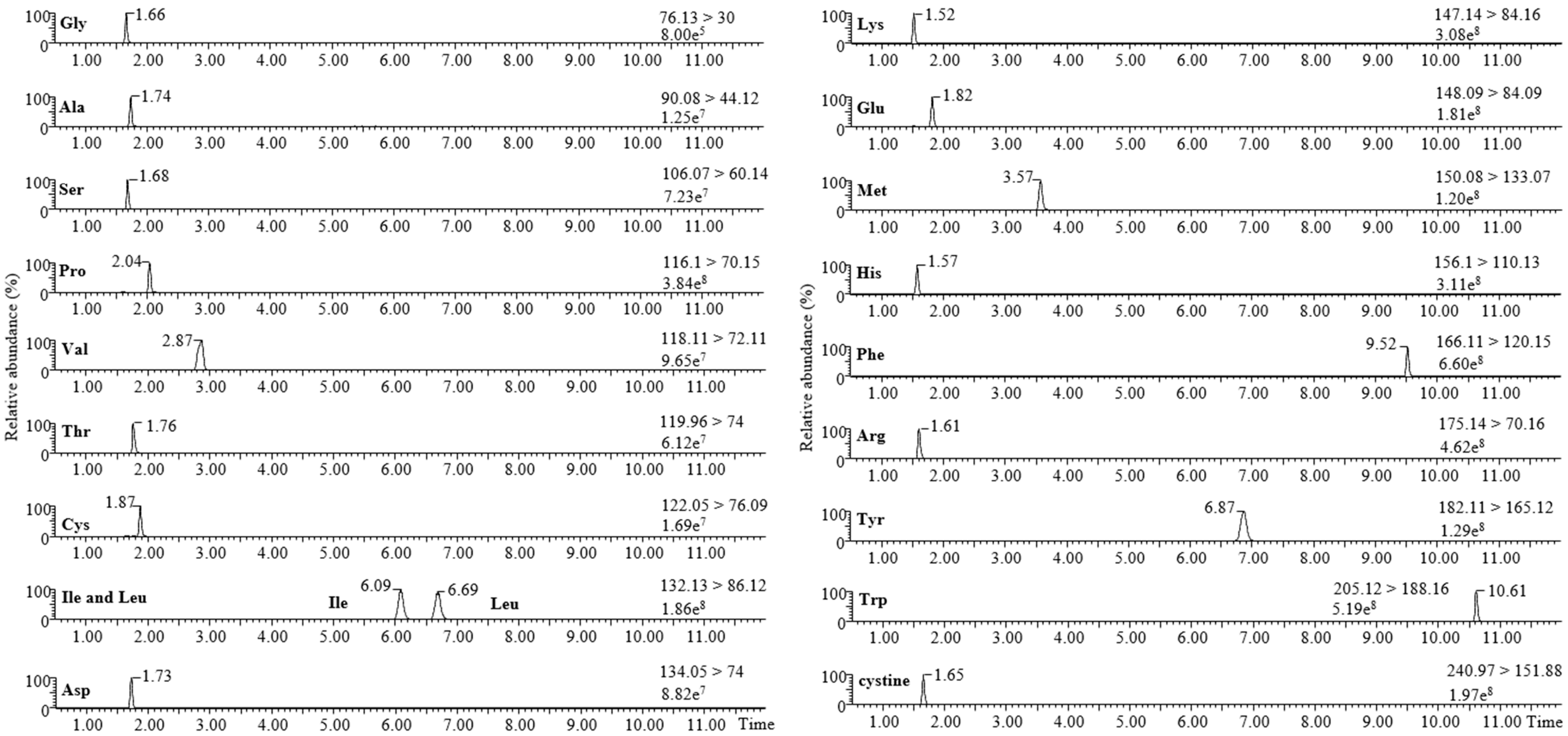

Figure S1. UHPLC-MS/MS chromatograms of amino acids in a mixed standard solution obtained by multiple reaction monitoring in positive ionization mode.

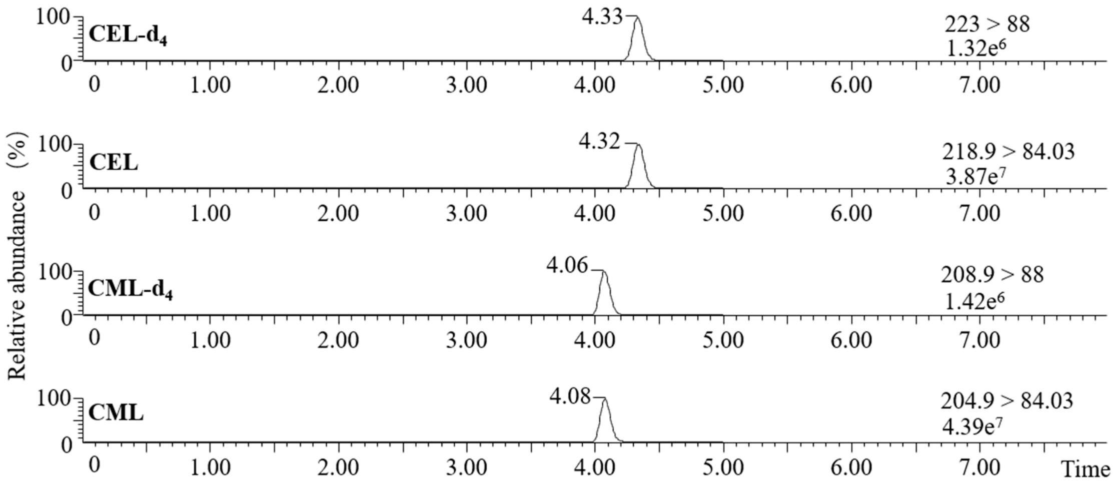

Figure S2. UHPLC-MS/MS chromatograms of CML, CEL and their respective internal standards in a mixed standard solution obtained by multiple reaction monitoring in positive ionization mode.

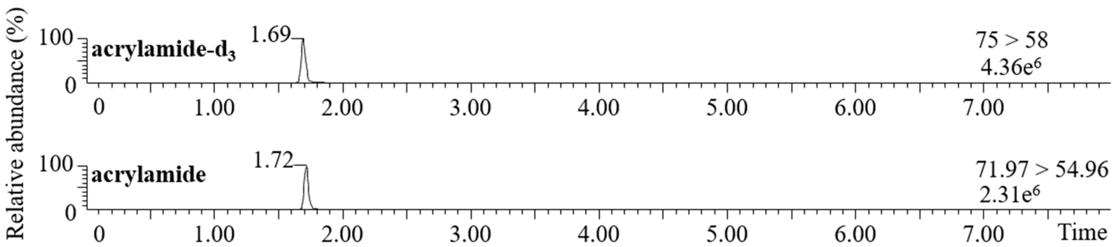

Figure S3. UHPLC-MS/MS chromatograms of acrylamide and its internal standard in a mixed standard solution obtained by multiple reaction monitoring in positive ionization mode.

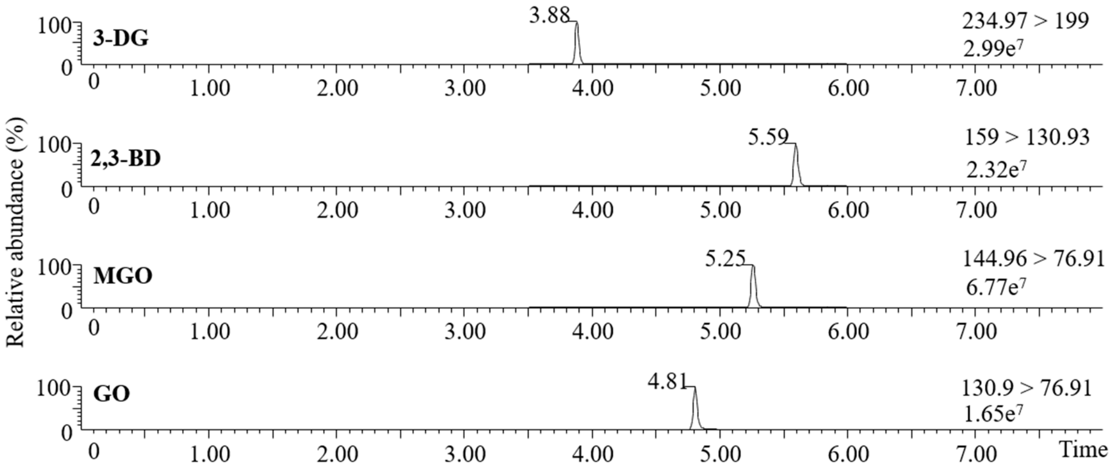

Figure S4. UHPLC-MS/MS chromatograms of α-dicarbonyl compounds (3-DG, 2,3-BD, MGO and GO) in a mixed standard solution obtained by multiple reaction monitoring in positive ionization mode.

Table S1. Content of amino acids in extrudates produced under different moistures.

	Amino acid (mg/kg)								
**Sample**	**Gly**	**Ala**	**Ser**	**Pro**	**Val**	**Thr**	**Leu**	**Ile**	**Asp**
20%	26.0 ± 2.3^{a}	30.9 ± 1.0^{ab}	21.1 ± 0.4^{b}	36.4 ± 0.7^{a}	33.8 ± 0.7^{a}	23.5 ± 0.5^{b}	56.1 ± 0.5^{bc}	32.2 ± 1.0^{a}	84.0 ± 0.2^{b}
30%	27.5 ± 1.4^{a}	29.6 ± 1.4^{b}	21.0 ± 0.2^{b}	36.3 ± 0.7^{a}	34.2 ± 0.8^{a}	23.1 ± 0.5^{b}	55.7 ± 0.5^{c}	32.7 ± 1.0^{a}	84.2 ± 1.3^{b}
40%	29.7 ± 2.0^{a}	30.6 ± 1.1^{ab}	21.8 ± 0.5^{ab}	37.4 ± 0.7^{a}	34.5 ± 1.2^{a}	24.2 ± 0.4^{ab}	57.6 ± 0.5^{ab}	33.2 ± 1.1^{a}	85.9 ± 1.6^{ab}
50%	29.7 ± 1.6^{a}	30.5 ± 1.3^{ab}	22.3 ± 0.6^{a}	37.4 ± 0.5^{a}	34.5 ± 0.5^{a}	24.3 ± 0.5^{ab}	57.5 ± 0.8^{ab}	33.1 ± 0.8^{a}	87.0 ± 0.2^{a}
60%	27.2 ± 0.8^{a}	32.6 ± 0.8^{a}	22.4 ± 0.2^{a}	37.4 ± 0.1^{a}	35.1 ± 0.7^{a}	25.4 ± 0.2^{a}	58.0 ± 0.3^{a}	34.0 ± 0.7^{a}	87.5 ± 0.9^{a}
raw material	29.3 ± 2.3^{a}	31.5 ± 0.3^{ab}	21.9 ± 0.4^{b}	37.0 ± 0.3^{a}	35.0 ± 1.0^{a}	23.7 ± 0.7^{bc}	57.5 ± 0.6^{ab}	34.0 ± 1.1^{a}	85.5± 0.7^{ab}
	**Amino acid (mg/kg)**								
**Sample**	**Lys**	**Glu**	**Met**	**His**	**Phe**	**Arg**	**Tyr**	**Trp**	**cystine**
20%	41.8 ± 0.6^{bc}	116.4 ± 0.6^{cd}	9.2 ± 0^{a}	15.8 ± 0.6^{a}	39.0 ± 0.6^{b}	69.1 ± 0.8^{bc}	28.0 ± 0.4^{b}	8.8 ± 0.3^{a}	5.6 ± 0.3^{c}
30%	43.0 ± 0.8^{abc}	115.5 ± 0.8^{d}	9.3 ± 0.3^{a}	16.1 ± 0.7^{a}	39.0 ± 0.2^{b}	68.1 ± 0.4^{c}	28.6 ± 0^{b}	8.3 ± 0.4^{a}	7.1 ± 0.2^{b}
40%	43.9 ± 1.1^{ab}	119.6 ± 2.1^{ab}	9.2 ± 0.3^{a}	16.6 ± 0.5^{a}	40.9 ± 0.7^{a}	70.6 ± 2.0^{abc}	29.8 ± 0.1^{a}	8.7 ± 0.2^{a}	7.5 ± 0.2^{ab}
50%	44.3 ± 1.1^{a}	119.2 ± 0.1^{abc}	8.8 ± 0.5^{a}	16.5 ± 0.7^{a}	40.9 ± 0.3^{a}	71.9 ± 0.7^{ab}	30.0 ± 0.3^{a}	8.8 ± 0.2^{a}	7.6 ± 0.2^{ab}
60%	43.4 ± 0.5^{abc}	121.6 ± 1.1^{a}	9.4 ± 0.5^{a}	16.2 ± 0.3^{a}	40.9 ± 0.4^{a}	73.5 ± 1.1^{a}	30.4 ± 0.3^{a}	8.8 ± 0.3^{a}	8.0 ± 0.3^{a}
raw material	41.3 ± 0.8^{c}	117.7 ± 0.9^{bcd}	9.6 ± 0.8^{a}	16.9 ± 0.8^{a}	41.1 ± 0.5^{a}	72.3 ± 1.3^{a}	30.4 ± 0.3^{a}	8.9 ± 0.3^{a}	7.9 ± 0.5^{a}

Raw material: soybean protein isolate: corn starch = 9:1. Values are statistically analyzed by one-way analysis of variance (ANOVA) with Tukey HSD test, and expressed as mean ± standard deviation (SD) (n = 3). Values in the same column marked by different letters indicate statistically different at 0.05 level.

Table S2. Content of amino acids in extrudates produced under different feed rates.

	Amino acid (mg/kg)								
**Sample**	**Gly**	**Ala**	**Ser**	**Pro**	**Val**	**Thr**	**Leu**	**Ile**	**Asp**
4 kg/h	30.3 ± 1.2^{a}	30.4 ± 0.7^{a}	21.0 ± 0.2^{a}	36.4 ± 0.4^{a}	34.5 ± 0.9^{a}	23.1 ± 0.5^{a}	56.2 ± 1.0^{a}	33.0 ± 1.1^{a}	83.7 ± 1.9^{a}
6 kg/h	27.5 ± 1.4^{a}	29.6 ± 1.4^{a}	21.0 ± 0.2^{a}	36.3 ± 0.7^{a}	34.2 ± 0.8^{a}	23.1 ± 0.5^{a}	55.7 ± 0.5^{a}	32.7 ± 0.9^{a}	84.2 ± 1.3^{a}
8 kg/h	26.4 ± 0.7^{a}	30.7 ± 0.1^{a}	21.3 ± 0.8^{a}	36.4 ± 0.7^{a}	35.0 ± 0.7^{a}	23.2 ± 0.8^{a}	56.2 ± 1.3^{a}	33.5 ± 0.4^{a}	83.9 ± 2.0^{a}
raw material	29.3 ± 2.3^{a}	31.5 ± 0.3^{a}	21.9 ± 0.4^{a}	37.0 ± 0.3^{a}	35.0 ± 1.0^{a}	23.7 ± 0.7^{a}	57.5 ± 0.6^{a}	34.0 ± 1.1^{a}	85.5 ± 0.7^{a}
	**Amino acid (mg/kg)**								
**Sample**	**Lys**	**Glu**	**Met**	**His**	**Phe**	**Arg**	**Tyr**	**Trp**	**cystine**
4 kg/h	41.8 ± 0.8^{a}	115.6 ± 2.4^{a}	9.3 ± 0.1^{a}	16.6 ± 0.5^{a}	40.0 ± 0.3^{ab}	69.9 ± 0.1^{b}	29.2 ± 0.7^{ab}	8.5 ± 0.2^{a}	6.8 ± 0^{b}
6 kg/h	43.0 ± 0.8^{a}	115.5 ± 0.8^{a}	9.3 ± 0.3^{a}	16.1 ± 0.7^{a}	39.0 ± 0.2^{b}	68.1 ± 0.4^{b}	28.6 ± 0^{b}	8.3 ± 0.4^{a}	7.1 ± 0.2^{b}
8 kg/h	41.7 ± 0.7^{a}	116.1 ± 3.6^{a}	9.4 ± 0.3^{a}	16.7 ± 0.3^{a}	40.3 ± 1.2^{ab}	70.4 ± 1.1^{ab}	29.6 ± 1.0^{ab}	8.7 ± 0.6^{a}	7.4 ± 0.2^{ab}
raw material	41.3 ± 0.8^{a}	117.7 ± 0.9^{a}	9.6 ± 0.1^{a}	16.9 ± 0.8^{a}	41.1 ± 0.5^{a}	72.3 ± 1.3^{a}	30.4 ± 0.3^{a}	8.8 ± 0.3^{a}	7.9 ± 0.5^{a}

Raw material: soybean protein isolate: corn starch = 9:1. Values are statistically analyzed by one-way analysis of variance (ANOVA) with Tukey HSD test, and expressed as mean ± standard deviation (SD) ($n = 3$). Values in the same column marked by different letters indicate statistically different at 0.05 level.

Table S3. Content of amino acids in extrudates produced under different screw speeds.

	Amino acid (mg/kg)								
**Sample**	**Gly**	**Ala**	**Ser**	**Pro**	**Val**	**Thr**	**Leu**	**Ile**	**Asp**
120 rpm	23.1 ± 1.4^{b}	28.7 ± 2.6^{a}	20.4 ± 0.8^{b}	34.5 ± 1.5^{b}	32.8 ± 1.7^{a}	21.7 ± 1.6^{a}	53.1 ± 2.6^{b}	31.0 ± 2.1^{a}	79.0 ± 4.1^{b}
150 rpm	27.5 ± 1.4^{ab}	29.6 ± 1.4^{a}	21.0 ± 0^{ab}	36.3 ± 0.7^{ab}	34.2 ± 0.8^{a}	23.1 ± 0.5^{a}	55.7 ± 0.5^{ab}	32.7 ± 1.0^{a}	84.2 ± 1.3^{ab}
180 rpm	28.3 ± 1.8^{a}	30.4 ± 0.7^{a}	21.6 ± 0.4^{ab}	36.0 ± 0.5^{ab}	32.7 ± 0^{a}	24.0 ± 1.2^{a}	55.1 ± 0.7^{ab}	31.7 ± 0.1^{a}	83.6 ± 0.5^{ab}
raw material	29.3 ± 2.3^{a}	31.5 ± 0.3^{a}	21.9 ± 0.4^{a}	37.0 ± 0.3^{a}	35.0 ± 1.0^{a}	23.7 ± 0.7^{a}	57.5 ± 0.6^{a}	34.0 ± 1.1^{a}	85.5 ± 0.7^{a}
	**Amino acid (mg/kg)**								
**Sample**	**Lys**	**Glu**	**Met**	**His**	**Phe**	**Arg**	**Tyr**	**Trp**	**cystine**
120 rpm	38.2 ± 2.9^{b}	109.8 ± 7.4^{a}	8.7 ± 0.5^{a}	15.9 ± 1.2^{a}	38.6 ± 2.0^{a}	67.6 ± 3.2^{c}	27.9 ± 1.5^{b}	8.8 ± 0.1^{a}	6.5 ± 0.1^{b}
150 rpm	43.0 ± 0.8^{a}	115.5 ± 0.8^{a}	9.3 ± 0.3^{a}	16.1 ± 0.7^{a}	39.0 ± 0.2^{a}	68.1 ± 0.4^{bc}	28.6 ± 0^{ab}	8.3 ± 0.4^{a}	7.1 ± 0.2^{ab}
180 rpm	39.7 ± 0.5^{ab}	117.6 ± 1.4^{a}	9.2 ± 0.3^{a}	16.8 ± 0.3^{a}	40.2 ± 0.3^{a}	72.9 ± 1.0^{a}	28.9 ± 0.2^{ab}	8.3 ± 0.1^{a}	6.6 ± 0.4^{b}
raw material	41.3 ± 0.8^{ab}	117.7 ± 0.9^{a}	9.6 ± 0.1^{a}	16.9 ± 0.8^{a}	41.1 ± 0.5^{a}	72.3 ± 1.3^{ab}	30.4 ± 0.3^{a}	8.9 ± 0.3^{a}	7.9 ± 0.5^{a}

Raw material: soybean protein isolate: corn starch = 9:1. Values are statistically analyzed by one-way analysis of variance (ANOVA) with Tukey HSD test, and expressed as mean ± standard deviation (SD) (n = 3). Values in the same column marked by different letters indicate statistically different at 0.05 level.

Table S4. Content of amino acids in extrudates produced under different barrel temperatures.

Sample	Amino acid (mg/kg)								
	**Gly**	**Ala**	**Ser**	**Pro**	**Val**	**Thr**	**Leu**	**Ile**	**Asp**
130℃	25.6 ± 1.6[a]	33.2 ± 0.8[a]	22.2 ± 0.2[a]	38.1 ± 0.2[a]	35.9 ± 0.4[a]	25.0 ± 1.3[a]	57.9 ± 0.8[a]	34.2 ± 0.7[a]	87.7 ± 0.7[a]
150℃	27.5 ± 1.4[a]	29.6 ± 1.4[b]	21.0 ± 0.2[b]	36.3 ± 0.7[b]	34.2 ± 0.8[ab]	23.1 ± 0.5[a]	55.7 ± 0.5[b]	32.7 ± 0.9[a]	84.2 ± 1.3[b]
170℃	28.8 ± 1.6[a]	31.5 ± 1.5[ab]	21.5 ± 0.2[ab]	36.8 ± 0.9[ab]	33.2 ± 0.7[b]	23.4 ± 0.1[a]	55.6 ± 1.2[b]	32.0 ± 0.9[a]	83.5 ± 1.8[b]
raw material	29.3 ± 2.3[a]	31.5 ± 0.3[ab]	21.9 ± 0.4[a]	37.0 ± 0.3[ab]	35.0 ± 1.0[ab]	23.7 ± 0.7[a]	57.5 ± 0.6[ab]	34.0 ± 1.1[a]	85.5 ± 0.7[ab]

Sample	Amino acid (mg/kg)								
	**Lys**	**Glu**	**Met**	**His**	**Phe**	**Arg**	**Tyr**	**Trp**	**cystine**
130℃	41.0 ± 0.3[b]	122.8 ± 3.4[a]	9.6 ± 0.3[a]	16.5 ± 0.3[ab]	42.1 ± 0.2[a]	75.5 ± 1.0[a]	30.9 ± 0.2[a]	9.4 ± 0.1[a]	7.4 ± 0.4[ab]
150℃	43.0 ± 0.8[a]	115.5 ± 0.8[b]	9.3 ± 0.3[a]	16.1 ± 0.7[a]	39.0 ± 0.2[c]	68.1 ± 0.4[c]	28.6 ± 0[c]	8.3 ± 0.4[b]	7.1 ± 0.2[ab]
170℃	40.4 ± 0.8[b]	116.4 ± 1.2[b]	8.8 ± 0.6[a]	17.2 ± 0.5[a]	40.3 ± 0.7[b]	72.2 ± 1.8[b]	29.4 ± 0.7[bc]	9.2 ± 0.2[a]	6.3 ± 0.3[b]
raw material	41.3 ± 0.8[ab]	117.7 ± 0.9[b]	9.6 ± 0.1[a]	16.9 ± 0.8[ab]	41.1 ± 0.5[ab]	72.3 ± 1.3[b]	30.4 ± 0.3[ab]	8.9 ± 0.3[ab]	7.9 ± 0.5[a]

Raw material: soybean protein isolate: corn starch = 9:1. Values are statistically analyzed by one-way analysis of variance (ANOVA) with Tukey HSD test, and expressed as mean ± standard deviation (SD) (n = 3). Values in the same column marked by different letters indicate statistically different at 0.05 level.

Table S5. Correlations of amino acids with CML, CEL, acrylamide, α-dicarbonyl compounds, protein, water in self-made plant based meat analogues produced under various extrusion parameters (n = 33).

**Parameter**	**Correlation coefficient (r)**														
	**Gly**	**Ala**	**Ser**	**Pro**	**Val**	**Thr**	**Leu**	**Ile**	**Glu**	**Met**	**His**	**Phe**	**Tyr**	**Trp**	**Cystine**
CEL	-0.065	-0.209	-0.494**	-0.359	-0.424*	-0.380*	-0.368*	-0.435*	-0.373*	-0.082	-0.128	-0.529**	-0.622**	-0.091	-0.846**
CML	-0.027	0.265	0.504**	0.345	0.324	0.373*	0.315	0.323	0.336	0.138	-0.119	0.364*	0.452*	0.137	0.763**
AGEs	-0.083	-0.178	-0.453*	-0.334	-0.415*	-0.352	-0.352	-0.428*	-0.353	-0.061	-0.18	-0.529**	-0.616**	-0.072	-0.800**
acrylamide	-0.012	-0.085	0.103	0.082	0.208	0.036	0.062	0.164	0.1	0.141	0.093	0.302	0.279	-0.021	0.453*
MGO	0.024	-0.132	-0.303	-0.248	-0.426*	-0.311	-0.376*	-0.418*	-0.282	-0.227	0.07	-0.293	-0.382*	0.135	-0.744**
GO	-0.076	0.392*	0.599**	0.432*	0.395*	0.585**	0.431*	0.405*	0.495**	0.137	0.02	0.518**	0.630**	0.273	0.711**
3-DG	0.062	0.375*	0.604**	0.438*	0.398*	0.573**	0.497**	0.446*	0.482**	0.145	-0.044	0.420*	0.541**	0.066	0.750**
2,3-BD	0.167	-0.074	-0.071	-0.162	-0.431*	-0.143	-0.291	-0.365*	-0.161	-0.291	0.086	-0.169	-0.22	0.222	-0.550**
Protein	-0.016	0.427*	0.653**	0.516**	0.399*	0.569**	0.498**	0.390*	0.508**	0.203	0.057	0.557**	0.626**	0.325	0.703**
Water	0.215	0.293	0.607**	0.463*	0.373*	0.542**	0.526**	0.432*	0.470**	0.064	-0.008	0.414*	0.523**	-0.005	0.770**

Correlation analysis is performed using two-tailed Pearson's correlation. *p*–Values were adjusted for multiple comparisons using the false discovery rate (FDR). *, FDR-adjusted *p* value < 0.05, compounds were significantly correlated at 0.05 level; **, FDR-adjusted *p* value < 0.01, compounds were significantly correlated at 0.01 level. AGEs: the sum of CML and CEL; MGO: methylglyoxal; GO: glyoxal; 3-DG: deoxyglucosone; 2,3-BD: 2,3-butanedione.

Table S6. Retention times and MS/MS parameters of amino acids by multiple reaction monitoring (MRM).

Amino acid	Retention time (min)	Precursor ion (m/z)	Product ion (m/z)	Cone voltage (v)	Collision energy (ev)
Ala	1.74	90.08	44.12*	18	6
			62	18	6
Gly	1.66	76.13	30*	12	6
			48	12	6
Ser	1.68	106.07	60.14*	18	8
			88.1	18	6
Pro	2.04	116.1	70.15*	18	12
			43	18	22
Val	2.87	118.11	72.11*	18	18
			55.1	18	8
Thr	1.76	119.96	74*	14	8
			56.08	14	14
			101.94	14	6
Cys	1.87	122.05	76.09*	22	12
			59.1	22	20
Leu	6.69	132.13	86.12*	20	10
			69.1	20	18
Ile	6.09	132.13	86.12*	20	10
			69.1	20	18
Asp	1.73	134.05	74*	14	12
			88	14	10
Lys	1.52	147.14	84.16*	8	14
			130.12	8	8
Glu	1.82	148.09	84.09*	20	14
			56	20	22
			129.97	20	6
Met	3.57	150.08	133.07*	16	8
			56.01	16	14
			104.09	16	10
His	1.57	156.1	110.13*	16	14
			83.05	16	20
Phe	9.52	166.11	120.15*	22	10
			103.1	22	24
Arg	1.61	175.14	70.16*	12	18
			60.13	12	14
Tyr	6.87	182.11	165.12*	22	8
			90.9	22	26
			136.1	22	12
Trp	10.61	205.12	188.16*	24	10
			146.12	24	16
cystine	1.65	240.97	151.88*	30	14
			73.91	30	28
			119.93	30	20

Product ion marked with * was used for quantification, the other product ion was used for confirmation.

Table S7. Retention times and MS/MS parameters of CML, CEL, acrylamide, their isotope-labeled internal standards, and α-dicarbonyl compounds by multiple reaction monitoring (MRM).

**Compounds**	**Retention time (min)**	**Precursor ion (m/z)**	**Product ion (m/z)**	**Cone voltage (V)**	**Collision energy (eV)**
CML	4.08	204.9	84.03*	4	16
			130	4	10
CML-d_4	4.06	209	88*	2	18
CEL	4.32	219	84.03*	2	20
			130.03	2	12
CEL-d_4	4.33	223	88*	2	18
acrylamide	1.72	71.97	54.96*	20	8
			44	20	10
acrylamide-d_3	1.69	75	58*	22	10
GO	4.81	130.9	76.91*	4	25
			103.9	4	20
MGO	5.25	144.96	76.91*	4	25
			91.9	4	25
			117.99	4	20
2,3-BD	5.59	159	130.93*	2	20
			117.9	2	20
			76.9	2	20
3-DG	3.88	234.97	199*	2	15
			145	2	20

Product ion marked with * was used for quantification, the other product ion was used for confirmation.
